# Deep vein thrombosis in acute respiratory distress syndrome caused by bacterial pneumonia

**DOI:** 10.1186/s12890-021-01632-1

**Published:** 2021-08-14

**Authors:** Na Cui, Song Mi, Chunguo Jiang, Wanlu Sun, Wenping Mao, Liming Zhang, Xiaokai Feng

**Affiliations:** 1 Department of Pulmonary and Critical Care Medicine, Beijing Chao-Yang Hospital, Capital Medical University, No. 8, Gongti South Road, Chaoyang District, Beijing, 100020 People’s Republic of China; 2 Beijing Institute of Respiratory Medicine, Beijing, 100020 People’s Republic of China

**Keywords:** Acute respiratory distress syndrome, Bacterial pneumonia, Deep vein thrombosis, Mechanical ventilation, Systemic inflammatory response syndrome

## Abstract

**Background:**

Acute respiratory distress syndrome (ARDS) is a clinical syndrome characterized by acute hypoxaemia, and few studies have reported the incidence of deep vein thrombosis (DVT) in direct ARDS caused by bacterial pneumonia. We performed a study to evaluate the prevalence, risk factors, prognosis and potential thromboprophylaxis strategies of DVT in these patients.

**Methods:**

Ninety patients were included. Demographic, and clinical data, laboratory data and outcome variables were obtained, and comparisons were made between the DVT and non-DVT groups***.***

**Results:**

Of the 90 patients, 40 (44.4%) developed lower extremity DVT. Compared with non-DVT patients, DVT patients had higher systemic inflammatory response syndrome (SIRS) scores, lower serum creatinine levels, higher D-dimer levels, and higher rates of sedative therapy and invasive mechanical ventilation (IMV). Multivariate analysis showed an association between the SIRS score (OR 3.803, *P* = 0.027), level of serum creatinine (OR 0.988, *P* = 0.001), IMV (OR 5.822, *P* = 0.002) and DVT. The combination of SIRS score, serum creatinine level and IMV has a sensitivity of 80.0% and a specificity of 74.0% for screening for DVT. The survival rate within 28 days after ARDS in the DVT group was significantly lower than that in the non-DVT group (*P* = 0.003). There was no difference in the prevalence of DVT between the 41 patients who received thromboprophylaxis and the 49 patients who did not receive thromboprophylaxis (41.5% vs 46.9%; *P* = 0.603).

**Conclusions:**

The prevalence of DVT is high in hospitalized patients with direct ARDS caused by bacterial pneumonia and may be associated with adverse outcomes. The current thromboprophylaxis strategies may need to be further optimized.

## Background

Deep vein thrombosis (DVT) and pulmonary embolism (PE), collectively referred to as venous thromboembolism (VTE), constitute a major global burden of disease [1]. Some studies have demonstrated an increased risk of VTE in intensive care units (ICUs) [2–4]. Patients with acute respiratory distress syndrome (ARDS) are at high risk of DVT, as they are susceptible to both general risk factors for VTE and those specific to critical illness, such as sedation, immobilization, insertion of central venous catheter (CVC) and mechanical ventilation (MV) [2–4], combined with a severe inflammatory response and hypercoagulable states [5–10].

The incidence of DVT in direct ARDS caused by bacterial pneumonia has not been investigated. We performed a single institutional study in patients with direct ARDS caused by bacterial pneumonia to identify the prevalence, risk factors, prognosis and potential thromboprophylaxis strategies of DVT in a Tertiary A hospital.

## Methods

### Study design and population

We retrospectively enrolled adult patients (≥ 18 years old) with direct ARDS (according to the Berlin definition) [11] caused by bacterial pneumonia admitted to the Department of Pulmonary and Critical Care Medicine, Beijing Chao-Yang Hospital, Capital Medical University, Beijing, China, from January 1, 2015, to June 30, 2020. Exclusion criteria included chronic respiratory failure defined as PaO_2_ < 60 mmHg on room air or PaO_2_/FiO_2_ < 300 according to arterial blood gas analysis, active malignant tumour, cerebral stroke, acute myocardial infarction, fracture of lower limb or hip, major operation defined as lasting longer than 45 min, major trauma, joint replacement for hip or knee and acute spinal cord injury within the past month. Patients with a survival time less than 3 days and patients without lower extremity venous compression ultrasound data were also excluded. All included patients were screened for DVT using lower extremity venous compression ultrasound scanning. If there was more than one ultrasound scan for a single patient, all the results were recorded. According to venous ultrasound scanning, patients were divided into a DVT group and a non-DVT group.

The study was approved by the ethics committee of the Beijing Chao-Yang Hospital, Capital Medical University (2020-ke-429) and was in accordance with the 1964 Helsinki Declaration and its later amendments or comparable ethical standards. Informed consent was exempted by the ethics committee of the Beijing Chao-Yang Hospital, Capital Medical University.

### Clinical data

We analysed the medical records of the patients admitted to Beijing Chao-Yang Hospital which included demographic information, clinical characteristics, vital signs, laboratory findings, treatment, complications, and outcomes that were collected and analysed by 2 analysts. We analysed the survival of all patients within 28 days after ARDS. For patients discharged within 28 days, we followed up their survival status after discharge by telephone.

### Ultrasound study

Bedside ultrasound examinations were performed using aportable colour ultrasound scanner (CX50, Philips Medical Systems, the Netherlands, equipped with an L12-3/S5-1 probe). Lower extremity venous compression ultrasound and echocardiographic data were obtained from the Picture Archiving and Communication System. The presence of pulmonary artery hypertension was evaluated by adding a tricuspid regurgitation pressure gradient to the estimated right atrial pressure [12].

### Definitions

ARDS was defined according to the Berlin definition [11]. According to the severity of hypoxemia, patients were divided into three groups [11]: (1) mild: 200 mmHg (1 mmHg = 0.133 kPa) < PaO_2_/FiO_2_ ≤ 300 mmHg with positive end expiratory pressure (PEEP) or continuous positive airway pressure (CPAP) ≥ 5 cmH_2_O (1 cmH_2_O = 0.098 kPa); (2) moderate: 100 mmHg < PaO_2_/FiO_2_ ≤ 200 mmHg with PEEP ≥ 5 cmH_2_O; and (3) severe: PaO_2_/FiO_2_ ≤ 100 mmHg with PEEP ≥ 5 cmH_2_O. Bacterial pneumonia, including community-acquired pneumonia and hospital-acquired pneumonia, was diagnosed according to the Clinical Practice Guidelines of the Infectious Diseases Society of America and the America Thoracic Society [13, 14]. Distal thrombosis was defined as thrombosis in the veins of the calf muscle and at least 1 branch of the 3 pairs of deep calf veins (anterior tibial vein, posterior tibial vein, or peroneal vein), and proximal thrombosis was defined as a thrombosis in the popliteal vein or above. Acute kidney injury was identified according to the Kidney Disease: Improving Global Outcomes (KDIGO) clinical practice guidelines: serum creatinine levels increased by ≥ 0.3 mg/dl (≥ 26.5 µmol/l) within 48 h or by 1.5 times baseline within seven days[15]. Cardiac injury was defined as the serum levels of cardiac troponin I above the upper limit of the reference. The Padua prediction score was defined according to the Barbar model [16]. The Wells score for DVT was defined according to the Di Nisio model [1]. The Caprini score was defined according to the updated Caprini Risk Assessment Model (2013 Version) [17]. We applied the systemic inflammatory response syndrome (SIRS) score and Acute Physiology and Chronic Health Evaluation (APACHE) II score to assess the severity of disease [18–21].

### Statistical analyses

Categorical variables were described as numbers and percentages (%) and continuous variables were described as the mean, standard deviation (SD), median, and interquartile range (IQR). The Shapiro–Wilk test was used to verify normality. Differences between the DVT and non-DVT groups were assessed by a 2-sample *t* test for normally distributed continuous variables, the Mann–Whitney *U* test for non-normally distributed continuous variables, and the χ^2^ or Fisher exact test for categorical variables. Univariate and multivariate logistic regression models were used to examine the risk factors associated with DVT. Receiver operating characteristic (ROC) analysis was performed to calculate the sensitivity and specificity of risk factors for screening for DVT. Survival curves were plotted using the Kaplan–Meier survival curves model and compared between patients with and without DVT by log-rank test. All statistical analyses were performed using SPSS version 23.0 (Statistical Package for the Social Sciences, Chicago, IL USA). All tests were 2-tailed; *P* < 0.05 was considered statistically significant.

## Results

**Fig. 1:**
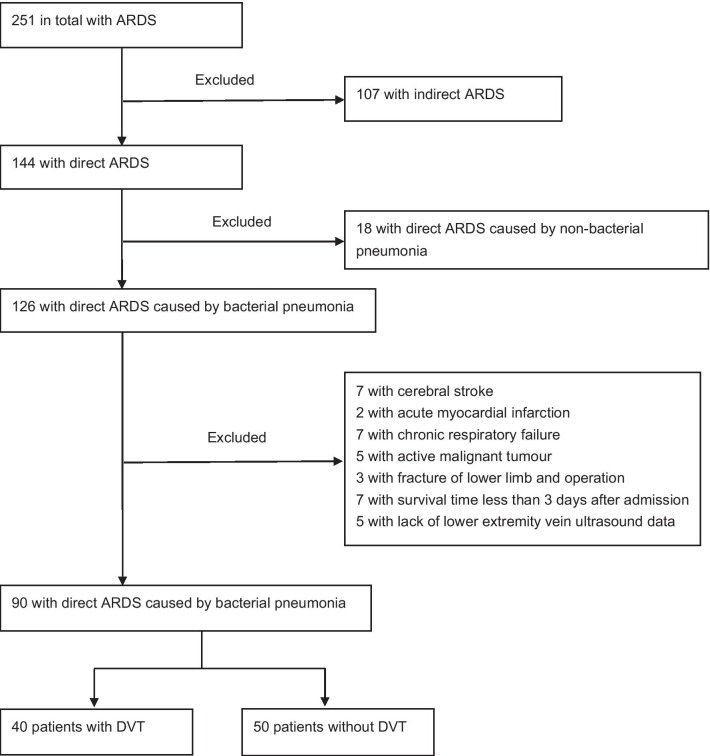
Flow chart of the study for including patients. ARDS, acute respiratory distress syndrome; DVT, deep vein thrombosis

**Fig. 2:**
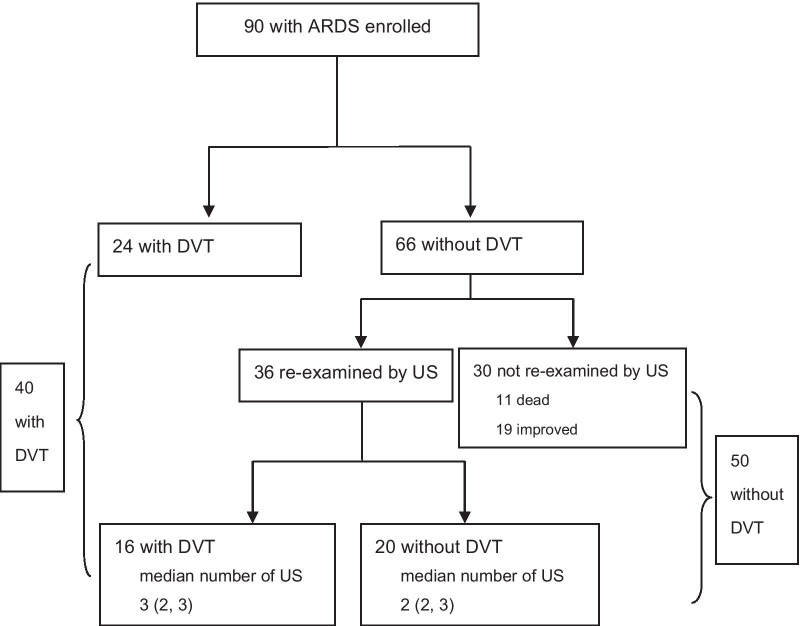
Flow chart of the study for screening DVT. ARDS, acute respiratory distress syndrome; DVT, deep vein thrombosis; US, ultrasonography

A total of 90 patients with direct ARDS caused by bacterial pneumonia were admitted to this study. The flow chart is shown in Figs. 1 and 2.

Some patients underwent more than one ultrasound scan. The median number of ultrasound examinations was 1 (range, 1–7). Of the 90 patients, 40 developed DVT and the prevalence of DVT was 44.4%, including 3 with proximal DVT and 37 with distal DVT, among which 30 had muscular calf vein thrombosis only. One patient was clinically suspected of PE, which was further confirmed by computed tomographic pulmonary angiography (CTPA) examination. The interval from the diagnosis of ARDS to the occurrence of DVT in the DVT group was 5 (2, 10) days, and the interval from the diagnosis of ARDS to the last ultrasound examination in the non-DVT group was 5 (2, 13) days. There were no differences between the two groups (*P* = 0.797).

Table 1 shows the demographic and clinical characteristics, laboratory data, treatments, complications and prognosis of our cohort. For all the patients, 68 received MV, among whom 54 (60.0%, 54/90) received IMV, 37 (41.1%, 37/90) received non-invasive mechanical ventilation (NIV), and 23 (25.6%, 23/90) received both IMV and NIV successively. Of the 90 patients, 41 (45.6%) were given VTE prophylaxis, for whom 35 (38.9%) received LMWH (25 received dalteparin 5000 IU once daily, 10 received nadroparin 0.1 ml/10 kg once daily), 4 (4.4%) received physical prevention only, 2 (2.2%) received other anticoagulation drugs such as warfarin (1 patient) or rivaroxaban (1 patient), and 26 (28.9%) received combined treatment with LMWH and physical prevention. Among the 49 patients who did not receive VTE prophylaxis, 14 had anticoagulant therapy contraindications, such as stress ulcers and gastrointestinal bleeding (6 patients), platelet counts less than 50 × 10^9^/L (6 patients), and heamoptysis (2 patients). The remaining 35(38.7%) patients had no clear high-risk factors for bleeding but did not receive VTE prophylaxis due to the actual clinical situation at that time in this retrospective observational study. For the 41 patients who received VTE prophylaxis, the incidence of DVT was 41.5% (17/41); however, for the patients who did not receive VTE prophylaxis, it was 46.9% (23/49), and there was no significant difference between the two groups (*P* = 0.603). For the 35 patients who received LMWH, the incidence of DVT was 40% (14/35), and for the 26 patients who received combined treatment with LMWH and physical prevention, it was 50.0% (13/26). There was no significant difference between the two groups (*P* = 0.437). In summary, compared with non-DVT patients, DVT patients had a higher proportion of SIRS score > 15 (47.5% vs 22.0%, *P* = 0.011), a lower level of serum creatinine (77.3 μmol/L vs 121.6 μmol/L, *P* = 0.006), a higher level of D-dimer (2.8 mg/L vs 1.9 mg/L, *P* = 0.035), a higher proportion of sedative therapy (57.5% vs 34.0%, *P* = 0.026) and a higher proportion of IMV (80.0% vs 44.4%, *P* = 0.001).

**Table 1:** Demographics, clinical characteristics, laboratory data, treatments, complications, and prognosis of DVT versus non-DVT patients with ARDS caused by bacterial pneumonia

Variables	Total (*n* = 90)	DVT (*n* = 40)	Non-DVT (*n* = 50)	*P* value
Age (years)	68 (57, 78)	71 (60, 80)	66 (51, 77)	0.150
Male, n (%)	69 (76.6)	30 (75.0)	39 (78.0)	0.738
BMI	23.8 ± 4.0	23.0 ± 3.9	24.4 ± 3.8	0.088
Bedridden time (days)	10 (6, 18)	9 (5, 19)	11 (7, 18)	0.603
CKD, n (%)	6 (6.7)	1 (2.5)	5 (10.0)	0.221
Wells score	1 (1, 1)	1 (1, 1)	1 (1, 2)	0.166
Padua prediction score	6 (5, 6)	6 (5, 6)	5 (5, 6)	0.481
Caprini score	7 (6, 9)	7 (5, 9)	7 (6, 9)	0.776
APACHE II score	22 (19, 28)	22 (18, 29)	23 (19, 28)	0.897
SIRS score	15 (13, 16)	15 (13, 17)	14 (13, 15)	0.016
≤ 15, n (%)	60 (66.7)	21 (52.5)	39 (78.0)	0.011^a^
> 15, n (%)	30 (33.3)	19 (47.5)	11 (22.0)	
*Coagulation function index*				
D-dimer (mg/L)	2.0 (0.9, 4.7)	2.8 (0.9, 6.1)	1.9 (0.7, 2.8)	0.035
PT (s)	13.9 (12.1, 16.0)	14.4 (12.2, 15.9)	13.5 (11.9, 16.2)	0.649
APTT (seconds)	32.3 (29.2, 36.0)	32.7 (29.0, 35.6)	31.9 (29.3, 36.9)	0.805
Scr (μmol/L)	89.8 (66.0, 194.6)	77.3 (56.4, 128.5)	121.6 (71.4, 235.5)	0.006
PAH, n (%)	32 (38.6)	15 (40.5)	17 (37.0)	0.739
PASP (mmHg)^b^	45.2 ± 13.1	50.2 ± 13.0	40.8 ± 11.9	0.041
*Treatments*				
CVC, n (%)	35 (38.9)	16 (40.0)	19 (38.0)	0.847
Sedative therapy, n (%)	40 (44.4)	23 (57.5)	17 (34.0)	0.026
CRRT, n (%)	10 (11.1)	3 (7.5)	7 (14.0)	0.502
HFNO, n (%)	5(5.6)	4(10.0)	1(2.0)	0.167
MV, n (%)	68 (75.6)	33 (82.5)	35 (70.0)	0.170
IMV, n (%)	54 (60.0)	32 (80.0)	22 (44.4)	0.001
Duration of IMV (days)	3 (2, 6)	4 (2, 6)	3 (2, 7)	0.927
NIV, n (%)	37 (41.1)	13 (32.5)	24 (48.0)	0.138
VTE prophylaxis, n (%)	41 (45.6)	17 (42.5)	24 (48.0)	0.603
LMWH, n (%)	35 (38.9)	14 (35.0)	21 (42.0)	0.498
LMWH + physical prophylaxis, n (%)	26 (28.9)	13 (32.5)	13 (26.0)	0.499
PaO_2_/FiO_2_	132 ± 60	122 ± 57	141 ± 61	0.141
Mild, n (%)	16 (17.8)	4 (10.0)	12 (24.0)	0.178^a^
Moderate, n (%)	40 (44.4)	18 (45.0)	22 (44.0)	
Severe, n (%)	34 (37.8)	18 (45.0)	16 (32.0)	
*Major complications*				
AKI, n (%)	46 (51.1)	17 (42.5)	29 (58.0)	0.144
ACI, n (%)	39 (43.3)	14 (35.0)	25 (50.0)	0.154
Coagulation dysfunction, n (%)	82 (91.1)	39 (97.5)	43 (86.0)	0.071
Septic shock, n (%)	39 (43.3)	18 (45.0)	21 (42.0)	0.775
ALI, n (%)	43 (47.8)	20 (50.0)	23 (46.0)	0.706
Number of US scan	1 (1, 2)	1 (1, 2)	1 (1, 2)	0.904
Duration to DVT or last negative US scan (days)	5 (2, 12)	5 (2, 10)	5 (2, 13)	0.797
Mortality, n (%)	38 (42.2)	18 (45.0)	20 (40.0)	0.633

Data are the mean ± SD, median (IQR) or n (%). *P* values comparing DVT and non-DVT were from the 2-sample *t* test, χ^2^ test, Fisher’s exact test or Mann–Whitney *U* test. *P* < 0.05 was considered statistically significant

ARDS, acute respiratory distress syndrome; DVT, deep vein thrombosis; BMI, body mass index; CKD, chronic kidney disease; APACHE II, Acute Physiology and Chronic Health Evaluation; SIRS, systemic inflammatory response syndrome; PT, prothrombin time; APTT, activated partial thromboplastin time; Scr, serum creatinine; PAH, pulmonary hypertension; PASP, pulmonary artery systolic pressure; CVC, central venous catheterization; CRRT, continuous renal replacement therapy; HFNO, high-flow nasal oxygen; MV, mechanical ventilation; IMV, invasive mechanical ventilation; NIV, non-invasive mechanical ventilation; VTE, venous thromboembolism; LMWH, low molecular weight heparin; PaO_2_, partial pressure of arterial oxygen; FiO_2_, fraction of inspired oxygen; Mild, 200 mmHg < PaO_2_ /FiO_2_ ≤ 300 mmHg; Moderate, 100 mmHg < PaO_2_/FiO_2_ ≤ 200 mmHg; Severe, PaO_2_ /FiO_2_ ≤ 100 mmHg; AKI, acute kidney injury; ACI, acute cardiac injury; ALI, acute liver injury; US, ultrasonic; IQR, interquartile range; SD, standard deviation

^a^χ^2^ test or Fisher’s exact test comparing all subcategories

^b^35.6% (32/90) of patients for whom PASP was available, with 15 patients in the DVT group and 17 patients in the non-DVT group

A total of 83 (92.2%) patients received echocardiogram examinations, with 37 patients in the DVT group and 46 patients in the non-DVT group. For the 32 (35.6%) patients with pulmonary artery hypertension, the DVT group had a higher level of pulmonary artery systolic pressure than the non-DVT group.

The results of univariate and multivariate logistic regression models are shown in Table 2. SIRS score, serum creatinine level, D-dimer level, sedative therapy and IMV were associated with DVT. Since all patients with sedation received IMV, there was a certain degree of overlap between these two variables, so we did not include sedative therapy in the multivariate regression. In the multivariate logistic regression model, we found that a higher SIRS score and lower levels of serum creatinine and IMV were associated with increased odds of DVT in patients with ARDS caused by bacterial pneumonia.

**Table 2:** Risk factors associated with DVT in patients with ARDS caused by bacterial pneumonia

Factors	Univariable OR (95% CI)	*P* value	Multivariable OR (95% CI)	*P* value
SIRS score				
≤ 15	1 (ref)		1 (ref)	
> 15	3.208 (1.288, 7.990)	0.012	3.803 (1.164, 12.422)	0.027
Scr (μmol/L)	0.993 (0.988, 0.998)	0.008	0.988 (0.981, 0.995)	0.001
D-dimer (mg/L)				
≤ 3.0	1 (ref)		1 (ref)	
> 3.0	3.043 (1.218, 7.602)	0.017	2.916 (0.925, 9.196)	0.068
Sedative therapy	2.626 (1.114, 6.191)	0.027		
IMV	5.091 (1.959, 13.230)	0.001	5.822 (1.877, 18.056)	0.002

ARDS, acute respiratory distress syndrome; DVT, deep vein thrombosis; SIRS, systemic inflammatory response syndrome; Scr, serum creatinine; IMV, invasive mechanical ventilation; OR, odds ratio; CI, confidence interval

*P* < 0.05 was considered statistically significant

Table 3 shows the demographics, clinical characteristics, laboratory data, treatments, complications, and prognosis of acute kidney injury (AKI) versus non-AKI patients of our cohort. Compared with non-AKI patients, AKI patients had higher SIRS score (*P* < 0.001), higher serum creatinine level (*P* < 0.001) and higher proportion of VTE prophylaxis (*P* = 0.010). There no difference in proportion of IMV between the two groups (*P* = 0.058).

**Table 3:** Demographics, clinical characteristics, laboratory data, treatments, complications, and prognosis of AKI versus non-AKI patients with ARDS caused by bacterial pneumonia

Variables	Total (*n* = 90)	AKI (*n* = 46)	Non-AKI (*n* = 44)	*P* value
Age (years)	68 (57, 78)	72 (59, 80)	66 (55, 76)	0.116
Male, n (%)	69 (76.6)	36 (78.3)	33 (75.0)	0.715
BMI	23.8 ± 4.0	23.6 ± 3.9	24.0 ± 4.1	0.606
CKD, n (%)	6 (6.7)	6 (13.0)	0 (0)	0.026
APACHE II score	22 (19, 28)	27 (22, 31)	19 (15, 23)	< 0.001
SIRS score	15 (13,16)	15 (14, 18)	13 (12, 15)	< 0.001
*Laboratory data*				
Scr (μmol/L)	89.8 (66.0, 194.6)	191.4 (123.4, 290.2)	66.9 (52.5, 78.4)	< 0.001
Ccr (mL/min)	55.9 (29.4, 97.6)	30.9 (20.5, 40.8)	98.2 (67.6, 118.2)	< 0.001
WBC (× 10^9^/L)	15.3 (11.6, 21.4)	17.4 (12.1, 23.0)	13.7 (11.2, 18.1)	0.033
Platelets (× 10^9^/L)	194 (126, 300)	180 (88, 255)	242 (143, 326)	0.014
Procalcitonin (ng/mL)	1.6 (0.4, 9.7)	5.4(1.2, 14.0)	0.6 (0.1, 2.6)	< 0.001
D-dimer (mg/L)	2.0 (0.9, 4.7)	2.3 (1.0, 4.9)	1.8 (0.6, 4.0)	0.155
PT (s)	13.9 (12.1, 16.0)	14.6 (12.2, 18.8)	13.4 (11.9, 15.5)	0.063
APTT (s)	32.3 (29.2, 36.0)	34.4 (29.6, 46.3)	31.0 (29.0, 33.7)	0.011
PaO_2_/FiO_2_	132 ± 60	120 ± 57	145 ± 60	0.046
*Treatments*				
CVC, n (%)	35 (38.9)	24 (52.2)	11 (25.0)	0.008
CRRT, n (%)	10 (11.1)	9 (19.6)	1 (2.3)	0.015
Sedative therapy, n (%)	40 (44.4)	26 (56.5)	14 (31.8)	0.018
IMV, n (%)	54 (60.0)	32 (69.6)	22 (50.0)	0.058
VTE prophylaxis, n (%)	41 (45.6)	27 (58.7)	14 (31.8)	0.010
LMWH, n (%)	35 (38.9)	22 (47.8)	13 (29.5)	0.075
LMWH + physical prophylaxis, n (%)	26 (28.9)	16 (34.8)	10 (22.7)	0.207
Physical prophylaxis only, n (%)	4 (4.4)	4 (8.7)	0 (0)	0.117
*Major complications*				
ACI, n (%)	39 (43.3)	27 (58.7)	12 (27.3)	0.003
Sepsis, n (%)	87 (96.7)	45 (97.8)	42 (95.5)	0.612
Septic shock, n (%)	39 (43.3)	27 (58.7)	12 (27.3)	0.003
ALI, n (%)	43 (47.8)	25 (54.3)	18 (40.9)	0.202
DVT, n (%)	40 (44.4)	17 (37.0)	23 (52.3)	0.144
Number of US scan	1 (1, 2)	2 (1, 3)	1 (1, 2)	0.023
Duration to DVT or last negative US scan (days)	5 (2, 12)	5 (1, 13)	6 (3, 11)	0.436
Mortality, n (%)	38 (42.2)	27 (58.7)	11 (25.0)	0.001

ARDS, acute respiratory distress syndrome; AKI, acute kidney injury; BMI, body mass index; CKD, chronic kidney disease; APACHE II, Acute Physiology and Chronic Health Evaluation; SIRS, systemic inflammatory response syndrome; Scr, serum creatinine; Ccr, creatinine clearance rate; WBC, white blood cells; PT, prothrombin time; APTT, activated partial prothrombin time; PaO_2_, partial pressure of arterial oxygen; FiO_2_, fraction of inspired oxygen; CVC, central venous catheterization; CRRT, continuous renal replacement therapy; IMV, invasive mechanical ventilation; VTE, venous thromboembolism; LMWH, low molecular weight heparin; ACI, acute cardiac injury; ALI, acute liver injury; DVT, deep vein thrombosis; US, ultrasonic

Data are the mean ± SD, median (IQR) or n (%). *P* values comparing AKI and non-AKI were from the 2-sample *t* test, Mann–Whitney *U* test, χ^2^ test, or Fisher’s exact test. *P* < 0.05 was considered statistically significant

Table 4 shows the renal function, coagulation parameters, SIRS score, IMV and DVT in AKI versus non-AKI patients stratified by VTE prophylaxis. For the 49 patients who did not receive VTE prophylaxis, compared with non-AKI patients, AKI patients had longer prothrombin time (PT; *P* = 0.014), longer activated partial prothrombin time (APTT; *P* = 0.027), higher SIRS score (*P* = 0.001) and higher proportion of IMV (*P* = 0.030). There was no difference in incidence of DVT between AKI patients and non-AKI patients (*P* = 0.962). For the 35 patients who received LWMH, the incidence of DVT in AKI patients was significantly lower than that in non-AKI patients (*P* = 0.046). There was no difference in PT, APTT, SIRS score and proportion of IMV between AKI patients and non-AKI patients.

**Table 4:** Renal function, coagulation parameters, SIRS score, IMV and DVT in AKI versus non-AKI patients with ARDS stratified by VTE prophylaxis

Variables	Non VTE prophylaxis (N = 49)			LMWH (N = 35)		
	AKI (n = 19)	Non-AKI (n = 30)	*P* value	AKI (n = 22)	Non-AKI (n = 13)	*P* value
Scr (μmol/L)	182.7 (119.7, 208.0)	67.9 (52.4, 80.4)	< 0.001	209.7 (145.8, 358.7)	66.1 (50.6, 78.0)	< 0.001
Ccr (mL/min)	32.2 (24.6, 45.3)	83.8 (61.9, 118.1)	< 0.001	27.3 (19.4, 41.0)	102.1 (78.5, 117.6)	< 0.001
PT (s)	15.9 (14.2, 22.5)	14.4 (11.9, 16.0)	0.014	13.3 (11.7, 15.7)	13.1 (11.3, 13.5)	0.347
APTT (s)	34.7 (30.5, 43.7)	30.8 (29.4, 33.6)	0.027	33.9 (27.6, 48.5)	32.1 (29.1, 34.5)	0.232
SIRS score	15 (14, 19)	13 (12, 15)	0.001	15 (13, 17)	13 (12, 16)	0.097
IMV, n %)	13 (68.4)	11 (36.7)	0.030	16 (72.7)	10 (76.9)	1.000
DVT	9 (47.4)	14 (46.7)	0.962	6 (27.3)	8 (61.5)	0.046

Ccr (mL/min) was estimated with the Cockroft-Gault equation: Ccr = ([140 – age in years] × body weight in kg)/(72 × serum creatinine in mg/dL). For women, the calculated values were multiplied by 0.85

ARDS, acute respiratory distress syndrome; AKI, acute kidney injury; Scr, serum creatinine; Ccr, creatinine clearance rate; PT, prothrombin time; APTT, activated partial prothrombin time; SIRS, systemic inflammatory response syndrome; IMV, invasive mechanical ventilation

Data are median (IQR) or n (%). *P* values comparing AKI and non-AKI were from Mann–Whitney *U* test, χ^2^ test, or Fisher’s exact. *P* < 0.05 was considered statistically significant

**Fig. 3:**
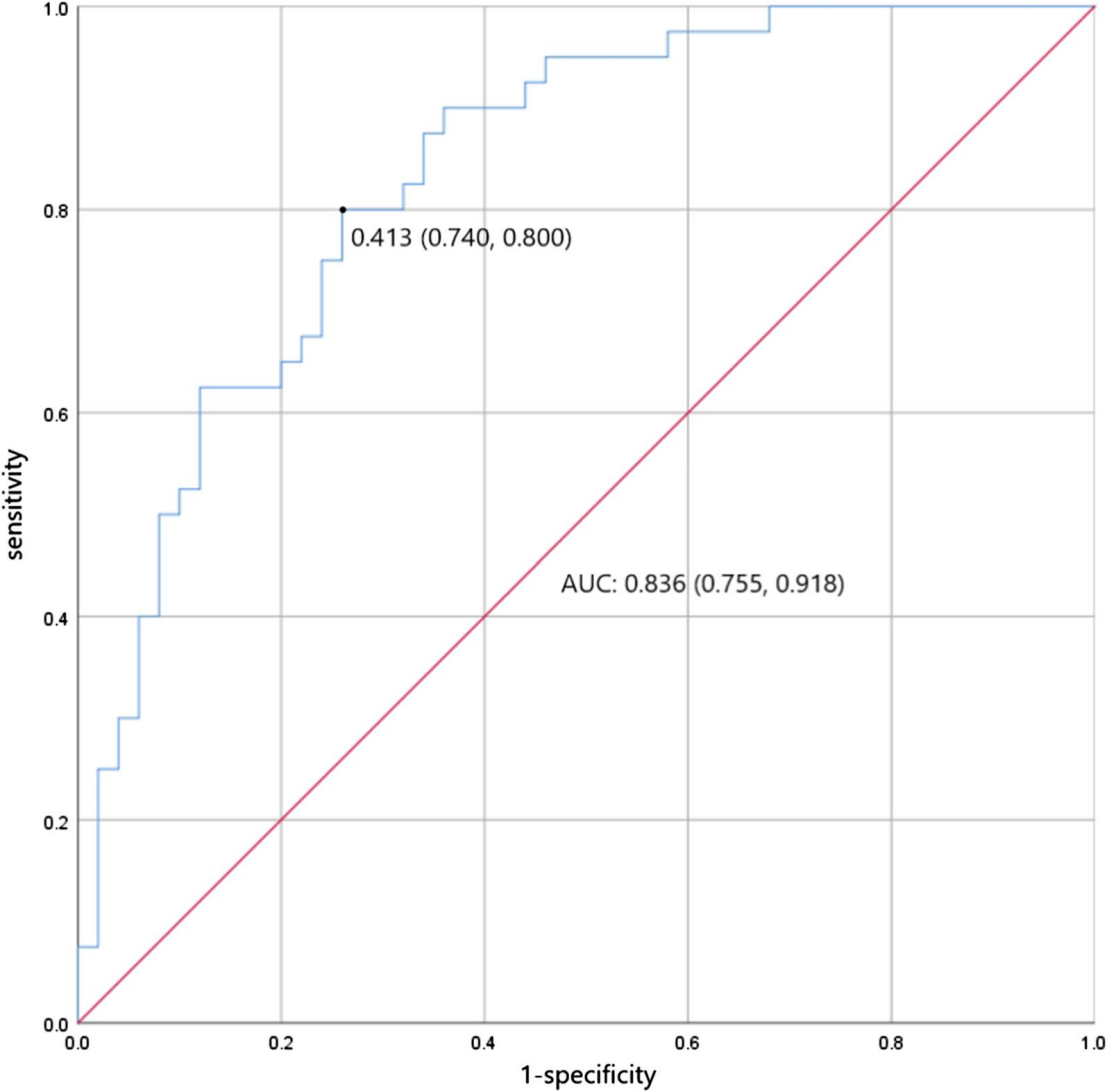
ROC curve for the diagnosis of DVT in ARDS patients caused by bacterial pneumonia with the combination of SIRS score, serum creatinine level and invasive mechanical ventilation. Multimodal features including SIRS score, serum creatinine level and invasive mechanical ventilation were incorporated into a single model (SSI model) for predicting DVT in patients with ARDS caused by bacterial pneumonia based on the multivariate logistic regression model. The final parameters in the equation are as follows: logit (DVT) =  − 1.620 + 0.255 × SIRS score − 0.013 × serum creatinine (μmol/L) − 1.856 × invasive mechanical ventilation. We generated the validated ROC curve based on the SSI model. Taking the prediction probability of 0.413 as the cut-off point, the model shows satisfactory forecasting ability for predicting DVT (AUC = 0.836; 95% CI: 0.755–0.918; sensitivity: 80.0%; specificity: 74.0%). ARDS, acute respiratory distress syndrome; DVT, deep vein thrombosis; ROC, receiver operating characteristic; AUC, area under the curve; CI, confidence interval

The combination of SIRS score, serum creatinine level and IMV showed the highest diagnostic accuracy for the prediction of DVT (area under the curve [AUC] = 0.836; 95% confidence interval [CI]: 0.755–0.918; sensitivity: 80.0%; specificity: 74.0%; *P* < 0.001) (Fig. 3).

**Fig. 4:**
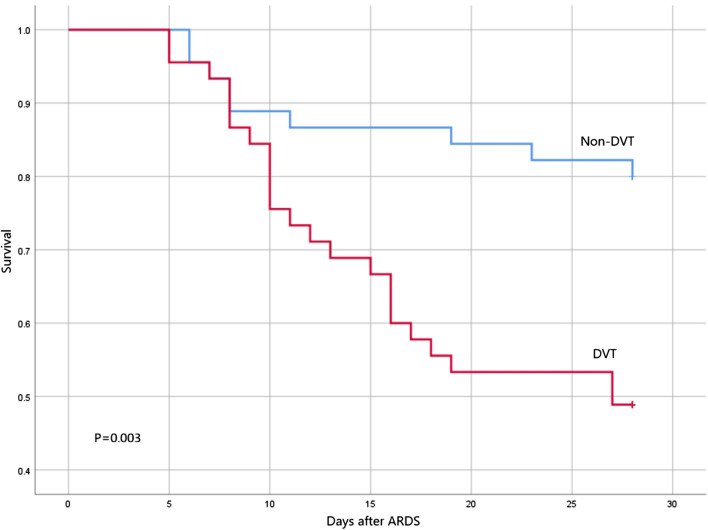
Kaplan–Meier survival curves showing the 28-day survival for patients with and without DVT in the ARDS cohort caused by bacterial pneumonia. (log-rank test). According to ROC curve, when the cut-off value of prediction probability is 0. 413, the Youden index is the highest, the model has a good prediction effect. For further verification, when *P* ≥ 0. 413 is the predicted DVT, the *P* < 0. 413 is the predicted non-DVT, and then the predicted DVT and non-DVT was used as a factor to construct Kaplan–Meier survival curve based on whether or not the patient died within 28 days. ARDS, acute respiratory distress syndrome; DVT, deep vein thrombosis; ROC, receiver operating characteristic

There was no difference in hospital mortality between the two groups. We further applied the Kaplan–Meier survival curves to analyse the survival rate within 28 days after ARDS and found that the survival rate in the DVT group was significantly lower than that in the non-DVT group (*P* = 0.003; Fig. 4).

## Discussion

In this single institutional study, we found a high prevalence of DVT on ultrasound scans and an association between DVT and SIRS score, serum creatinine level and IMV in these patients.

In 2002, Greets et al. reported that the rates of objectively confirmed DVT in 4 prospective studies ranged from 13 to 31% and suggested a potential role of thromboprophylaxis in patients requiring critical care [22]. In recent years, some research has shown that, despite receiving guideline-recommended thromboprophylaxis, the incidence of DVT is still as high as 14–37.2% in critically ill patients [2–4].

We studied 90 patients diagnosed with ARDS caused by bacterial pneumonia and the prevalence of DVT was 44.4% (40/90 studied). Several reasons probably account for the high prevalence of DVT in our study. First, most of the above studies generally focused on critically ill patients. ARDS is a more serious type of critical illness that shows an overwhelming systemic inflammatory process accompanied by an abnormal blood coagulation mechanism, and may have a higher risk of DVT [5–10]. Multiple studies have also suggested that the incidence of DVT in ARDS patients with coronavirus disease 2019 (COVID-19) or influenza A H_1_N_1_ is as high as 42.2–85.4% [23–25]. These conditions indicate that direct ARDS itself may be a risk factor for DVT. Second, some research defined VTE as pulmonary embolism, proximal DVT, and/or symptomatic distal DVT excluding asymptomatic isolated distal DVT [3].

Our study shows that SIRS score, serum creatinine level, D-dimer level, sedative therapy and IMV are related to the occurrence of DVT. Further multivariate regression analysis showed that the SIRS score, serum creatinine level and IMV were independent risk factors for DVT. Using a ROC analysis, the combination of SIRS score, serum creatinine level and IMV yielded a sensitivity of 80.0% and a specificity of 74.0% for scanning for DVT in these hospitalized patients.

Our research suggests that SIRS score > 15 points is an independent risk factor for DVT. Experimental and morphological studies suggest that inflammation and platelet activation also participate in DVT [26, 27]. A relationship between inflammation and thrombosis has been identified in different clinical scenarios where the inflammatory process and coagulation abnormalities are clearly interlinked. D-dimer is a molecular marker that results from the dissolution of cross-linked fibrin and is often elevated in thrombotic conditions. Our study found that the D-dimer level of DVT patients was significantly higher than that of non-DVT patients. However, there was no significant difference in the D-dimer level in multivariate regression. This may be because ARDS is a serious inflammatory response disease accompanied by obvious coagulation and fibrinolytic dysfunction [3, 28–31]. Similar to thrombotic diseases, it is also manifested as a significant increase in D-dimer levels, which may underestimate the effect of venous thrombosis on D-dimer levels.

In our research, the serum creatinine level of non-DVT patients was significantly higher than that of DVT patients, suggesting that patients with renal insufficiency may have a lower risk of DVT. Until now, the relationship between renal function and VTE is not clear. Some studies have shown that AKI and CKD are independent risk factors for VTE [32, 33]. However, Al-Dorzi et al. pointed out that, for critically ill patients, neither AKI nor end-stage renal disease was an independent risk factor for VTE [34]. In our cohort, 46 patients (51.1%) had AKI. We compared the clinical characteristics of AKI patients and non-AKI patients in detail, which showed that the proportion of receiving VTE prophylaxis was higher in patients with AKI. Further stratified analysis showed that, for the 49 patients who did not receive VTE prophylaxis, the incidence of DVT in AKI patients and in non-AKI patients had no significant difference. For these respondents, compared with patients without AKI, patients with AKI had significant exogenous and endogenous coagulation dysfunction (manifested by prolonged PT and APTT), which may lead to decreased risk of thrombosis. In addition, patients with AKI also had higher SIRS scores and a higher proportion of IMV, which might increase the risk of thrombosis. The combined effect of these factors led to no difference in the incidence of DVT between the two groups. For the 35 patients who received LWMH, the incidence of DVT in patients with AKI was significantly lower than that in patients without AKI. In this group, there was no significant difference in PT, APTT, SIRS score and proportion of IMV between patients with AKI and patients without AKI. Some studies have shown that LWMH may have different levels of bioaccumulation in the case of renal insufficiency [35–38]. The study by Cook et al*.* indicated that the incidence of DVT for patients with renal insufficiency in ICU who received dalteparin 5000 IU once daily was 5.1% [39], which was far lower than that in the overall population of critically ill patients who received preventive treatment recommended by the guidelines [2–4]. So we speculate that the same dose of LWMH may play a stronger role in the prevention of DVT in the case of renal insufficiency. Unfortunately, due to the retrospective nature of the study, the decrease of LWMH metabolism in patients with AKI and higher level of serum creatinine was based on the conjecture of clinical data analysis, and we did not detect the activity of anti-factor Xa. For the overall cohort, there was no difference in the incidence of DVT between patients with AKI and those without AKI. The possible reason is that for patients with AKI, an increase in the SIRS score as a procoagulant factor was present, together with a prolongation in APTT and a decrease in creatinine clearance, which were protective factors against thrombus formation, finally, the procoagulant factors may partially counteracted the antithrombotic factors.

ARDS is a clinical syndrome with a high mortality that usually requires MV, especially IMV [11]. In the case of IMV, sedation and immobilization are often performed simultaneously, which would aggravate blood stasis and increase the risk of DVT. Ren et al. proposed that IMV is a risk factor for DVT [23]. Knudson et al. pointed out that IMV longer than 3 days is an independent risk factor for VTE [40]. As the duration of IMV increases, the risk of DVT increases [3]. The study shows that IMV is an independent risk factor for DVT. However, in our study, compared with patients in the non-DVT group, the duration of IMV in the DVT group did not increase significantly, possibly because our small number of cases resulted in no statistically significant difference. On the other hand, some patients underwent lower extremity venous ultrasound at the early stage of ARDS once only and did not undergo sequential ultrasound monitoring, which led to underestimation of the incidence of subsequent DVT and the correlation between MV duration and DVT.

Similar to some previous studies [24, 41], our research shows that DVT is associated with adverse outcomes in these patients. The survival rate within 28 days after ARDS in the DVT group was significantly lower than that in the non-DVT group (*P* = 0.007).

Some studies have shown that for critically ill patients, accepting the preventive measures recommended by the guidelines can reduce the risk of DVT [2, 24, 42, 43]. However, many studies have also shown that even if the preventive measures recommended by the guidelines are taken, the risk of DVT is still high for critically ill patients, especially ARDS patients [3, 4, 23]. In our study, the overall incidence of DVT in ARDS patients was 44.4%. Further analysis showed that there was no difference in the incidence of DVT between patients who received VTE prophylaxis and patients who did not receive prophylaxis, and there was no difference in the incidence of DVT between patients who received LMWH and patients who received LMWH combined with physical prophylaxis. The reason may be that ARDS is a critical illness with a severe inflammatory response and is often accompanied by high-risk factors for VTE such as IMV, CVC insertion, sedation and immobilization. VTE cannot be effectively controlled by the prophylaxis recommended by the guidelines. This also suggests that for ARDS, thromboprophylaxis strategies may need to be strengthened, including moderately increasing the dose of anticoagulant drugs, especially for patients without renal insufficiency, or finding anti-inflammatory treatment targets to reduce the occurrence of DVT.

Our study first proposed a negative correlation between serum creatinine and DVT. However, there are some limitations in this study. First, due to the retrospective nature of the study, we did not detect coagulation indicators such as anti-factor Xa, protein C, and protein S, so the exact mechanism of the correlation between serum creatinine level and DVT needs further study. Second, the sample size of the study was small, which may underestimate the influence of factors such as age, obesity, and oxygenation on DVT. Third, as a retrospective observation, the time and frequency of ultrasound examination were not unified, and 35 (38.7%) patients who had no clear high-risk factors for bleeding but did not receive VTE prophylaxis due to the complexly clinical situation at that time, these were also deficiencies of this study. Finally, due to the critical condition of ARDS patients, CTPA examination was restricted. We only performed CTPA examination on 1 patient with highly suspected PE and confirmed the diagnosis of PE, which significantly underestimated the incidence of PE.

## Conclusion

In hospitalized patients with direct ARDS caused by bacterial pneumonia, the prevalence of DVT is high and is associated with adverse outcomes. We also found an association between DVT and multiple risk factors, especially SIRS score, serum creatinine level and IMV. A combination of the SIRS score, serum creatinine level and IMV provided a sensitivity of 80.0% and a specificity of 74.0% for screening for DVT. The current preventive measures may need to be further optimized to reduce the occurrence of DVT in ARDS.

## Data Availability

The anonymous dataset is available from the corresponding author.

## Abbreviations

ARDS: Acute respiratory distress syndrome
VTE: Venous thromboembolism
DVT: Deep vein thrombosis
PE: Pulmonary embolism
MV: Mechanical ventilation
IMV: Invasive mechanical ventilation
NIV: Non-invasive mechanical ventilation
AKI: Acute kidney injury
CKD: Chronic kidney disease
BMI: Body mass index
SIRS: Systemic inflammatory response syndrome
APACHE II: Acute Physiology and Chronic Health Evaluation
CVC: Central venous catheter
ICU: Intensive care unit
CTPA: Computed tomographic pulmonary angiography
PaO_2_: Partial pressure of arterial oxygen
FiO_2_: Fraction of inspired oxygen
PEEP: Positive end-expiratory pressure
CPAP: Continuous positive airway pressure
COVID-19: Coronavirus disease 2019
LMWH: Low-molecular-weight heparin
PT: Prothrombin time
APTT: Activated partial prothrombin time
